# Effect of light and nutrient availability on the release of dissolved organic carbon (DOC) by Caribbean turf algae

**DOI:** 10.1038/srep23248

**Published:** 2016-03-22

**Authors:** Benjamin Mueller, Joost den Haan, Petra M. Visser, Mark J. A. Vermeij, Fleur C. van Duyl

**Affiliations:** 1 Royal Netherlands Institute for Sea Research (NIOZ) Department of Marine Microbiology and Biogeochemistry and Utrecht University, P.O. Box 59, 1790AB Den Burg, Texel, The Netherlands; 2 CARMABI Foundation, Piscaderabaai z/n, P.O. Box 2090, Willemstad, Curaçao; 3 University of Amsterdam, Department of Aquatic Microbiology, P.O. BOX 94248, 1090 GE Amsterdam, The Netherlands; 4 Max-Plank Institute for Marine Microbiology (MPI Bremen), Celsiusstr. 1, 28359 Bremen, Germany

## Abstract

Turf algae increasingly dominate benthic communities on coral reefs. Given their abundance and high dissolved organic carbon (DOC) release rates, turf algae are considered important contributors to the DOC pool on modern reefs. The release of photosynthetically fixed carbon as DOC generally, but not always, increases with increased light availability. Nutrient availability was proposed as an additional factor to explain these conflicting observations. To address this proposed but untested hypothesis, we documented the interactive contributions of light and nutrient availability on the release of DOC by turf algae. DOC release rates and oxygen production were quantified in incubation experiments at two light levels (full and reduced light) and two nutrient treatments (natural seawater and enriched seawater). In natural seawater, DOC release at full light was four times higher than at reduced light. When nutrients were added, DOC release rates at both light levels were similar to the natural seawater treatment at full light. Our results therefore show that low light in combination with low nutrient availability reduces the release of DOC by turf algae and that light and nutrient availability interactively determine DOC release rates by this important component of Caribbean reef communities.

The concentration and composition of waterborne dissolved organic carbon (DOC) plays an important role in the functioning of coral reef ecosystems (e.g.^1^,^2^,^3^,^4^). The DOC pool on coral reefs is mainly fueled by benthic primary producers (i.e., benthic algae, benthic cyanobacterial mats and scleractinian corals)^5^,^6^,^7^,^8^, which release a substantial part of their photosynthetically fixed carbon as DOC into the water column^7^,^9^,^10^. Benthic algae generally release more DOC per surface area than corals (e.g.^7^,^10^,^11^) and algal DOC can promote the growth of opportunistic microbes in the interface of coral-algal interactions (reviewed in^3^). The combined effects of a shift in the microbial community towards opportunistic pathogens^12^,^13^, an increase in microbial abundance and respiration^7^,^14^ and as a result oxygen depletion^15^,^16^,^17^ can lead to mortality of the coral^18^,^19^,^20^ and therefore negatively influences the outcome of coral-algal competition^3^,^21^. The global increase of benthic algae will thus likely come with major implications for the DOC dynamics and the general functioning of coral reefs^1^,^7^,^10^,^22^.

Of all benthic algal groups, the abundance of turf algae has increased most dramatically in recent decades and turf algae presently represent the most dominant benthic component on many coral reefs around the world^15^,^21^,^23^,^24^,^25^. Turf algae are multi-species assemblages of Chlorophyta, Phaeophyta and Rhodophyta intermixed with filamentous cyanobacteria with a maximum height of approximately 1 cm and a distinct community of associated bacteria^26^,^27^,^28^. They exhibit fast growth rates^29^, rapidly take up nutrients^30^ and are capable of nitrogen fixation due to the presence of cyanobacteria within these communities^31^,^32^. Moreover, their high surface to volume ratio facilitate the exchange of metabolic products (e.g., DOC) between these algal communities and their environment^33^ and makes them one of the most productive benthic primary producers on coral reefs^34^,^35^. Given their abundance and high DOC release rates turf algae can be considered important contributors to the local DOC pool on modern coral reefs 7,8,10.

Light (e.g.^36^,^37^,^38^) and nutrient availability^37^,^39^ influence DOC release rates of aquatic primary producers. Potential interactive effects among these factors are less often considered and could underlie the sometimes contradicting observations of DOC release studies. For example, many authors have reported on the positive effect of light availability on DOC release^37^,^39^,^40^,^41^, whereby carbon fixation is hypothesized to outpace cell growth, particularly under nutrient limited conditions (e.g.^37^,^38^,^39^). Other authors were unable to confirm such a positive relationship between DOC release and light availability (e.g.^42^,^43^,^44^,^45^) and instead suggested that DOC passively diffuses through the cell membrane, independent of light availability. These contrasting observations are certainly not mutually exclusive^46^,^47^ and suggest that actual DOC release rates likely depend on more than one environmental condition (e.g., light and nutrient availability).

Light availability alone or in combination with increased nutrient availability due to eutrophication (e.g.^48^,^49^) could affect the rate of DOC release by Caribbean turf algae according to the light-dependent and/or light-independent mechanisms described by Carlson^46^. The purpose of this study was to determine if DOC release by turf algae depends on (1) light availability and/or (2) nutrient availability and if so (3) whether the contribution of light and nutrient availability is interactive or additive.

## Materials and Methods

### Experimental set up

The study was conducted in May 2012 on Curaçao, an island in the Southern Caribbean, 65 kilometers north of Venezuela. Fringing reefs run along its entire leeward coast^50^. Following the protocol of Den Haan, *et al*.^32^, turf algae were grown on the exterior of 0.5 L polyethylene (PET) bottles that were placed inside a 1 m^3^ chicken-wire cage (mesh size: 2.5 cm) to minimize grazing by larger herbivorous fish. The cage was deployed on the fore reef slope at 10 m depth at the site ‘Buoy 0’ (12°12′35″ N, 68°97′10″), which is located 500 m downstream of the outlet of the eutrophied Piscadera Bay. The benthic community at this site was dominated by macroalgae, turf algae and benthic cyanobacteria and hard coral cover is only 10%^51^. After 6 weeks, turf communities, representative of those growing on the reef bottom, had developed on the bottles^27^,^51^. Bottles covered with turf algae were collected 24 h prior to the experiment and the bottom and top part of the bottles were cut off. The resulting open cylinders (height = 8 cm, surface area = 282.4 ± 1.1 cm^2^ [mean ± SE]) were allowed to recover and acclimatize in flow-through seawater aquaria (27–29 °C) for at least 24 h. During this acclimatization period, light conditions were on average ~100 μmol photons m^−2 ^s^−1^ during the daytime as measured with a Hydrolab DS5 (OTT Messtechnik GmbH & Co., Kempten, Germany; sampling interval 30 s), which is similar to the light conditions measured at 20 m depth at Buoy 0 at midday^32^. Remaining bottles were completely scraped to serve as controls (PET without turf algae) for all experiments.

Turf algae were incubated in transparent Plexiglas incubators (1.0 L) with an opaque bottom and lid. The lid had a removable (Ø 5 cm) PVC plug to allow water sampling and a magnetic stirrer to ensure mixing throughout the experiment. Prior to the experiments, incubators were acid-washed (0.4 M HCl) and rinsed twice with filtered treatment water (0.22 μm Whatman Cellulose acetate membrane filter) to remove phytoplankton and planktonic microbes that could release or consume DOC. Incubators were filled with 1.0 L of filtered treatment water and turf algae (n = 4 per treatment) or a control cylinder (n = 1 per treatment) were subsequently placed in the incubators. Two nutrient treatments were used: (1) natural seawater and (2) nutrient enriched seawater. Enriched seawater was prepared 1 h prior to the experiments by adding nutrients in the form of NH_4_Cl, NaNO_3_ and KH_2_PO_4_ (Sigma Aldrich) to filtered seawater (Table 1).

Resulting nutrient concentrations (23 times dissolved inorganic nitrogen and 171 times PO_4_^3−^ concentration compared to those of the natural seawater treatment) were not intended to mirror naturally occurring concentrations, but rather to create nutrient replete conditions during the course of the incubations. Furthermore, these concentrations are similar to those used in nutrient uptake experiments and were proven to be non-lethal for the used turf algal communities^30^. The incubators were placed in a flow-through seawater system to keep them at temperatures similar to those on the reef (27–29 °C). During the 6 h experiments (all conducted between 10:00 hrs and 16:00 hrs local time, to ensure sufficient light levels during the entire duration of the experiments), incubators were exposed to one of two light treatments: (1) “full light” and (2) “reduced light”. In the “full light” treatment, incubators were placed in full sunlight receiving an average light intensity of 631 ± 52 μmol photons m^−2 ^s^−1^ (±SD). In the “reduced light” treatment incubators were wrapped in Neutral Density Filter shading foil (Modulor GmbH, Berlin, Germany) reducing the light intensity inside the incubators by 83% to an average of 105 ± 18 μmol photons m^−2 ^s^−1^ (Table 1).

At the beginning and end of the 6 h incubation, a water sample (60 mL) was taken from each incubator for DOC analysis using a polypropylene syringe (100 mL) that was acid-washed and rinsed with filtered treatment water beforehand. Samples were immediately placed in the dark and processed within 60 minutes after sampling. Additional water samples were taken at the beginning of the experiment to determine the initial nutrient concentrations in each incubator using a 50 mL Terumo syringe. Water samples were immediately filtered using 0.22 μm Acrodisc filters and stored in 6 mL polyethylene vials (PerkinElmer, MA, USA) at −20 °C until further analysis. Oxygen concentrations in each incubator were measured at hourly intervals during the duration of the experiment using an oxygen optode (PreSens Fibox 3). Oxygen production was determined to serve as a proxy for carbon fixation and to express DOC release as percentage of primary production (e.g.^7^,^8^). PH was measured at the beginning and the end of the experiment with a pH meter (WTW pH 330). The variability in the amount of light entering the incubation chambers during the experiment was quantified using a Hydrolab DS5 (OTT Messtechnik GmbH & Co., Kempten, Germany; sampling interval 30 s) that was standing in the direct vicinity of the incubators. Obtained light values by the Hydrolab were transformed to light values that would have occurred inside the incubators using a pre-determined conversion factor. This conversion factor was obtained by measuring the light intensity inside and outside of the incubators, with and without shading foil, using a light meter (cosine LI-192SSA underwater quantum sensor connected to LI-1000 data logger; range: PAR 400–700). The two light and nutrient treatments were combined in a factorial sampling design and two light-nutrient combinations were simultaneously run per day, in which we ensured that abiotic variables were comparable amongst sampling days (see Table 1). After the experiment, turf algae were scraped off each open cylinder to determine their dry weight (DW). Samples were rinsed with distilled water, oven-dried to constant weight in pre-weighed aluminum cups (>72 h at 60 °C) and weighed (accuracy 0.001 g). The mean DW of turf algae per cylinder was 0.396 ± 0.149 g (±SD).

### DOC and nutrient analyses

DOC samples were filtered (<20 kPa Hg suction pressure) over a 0.2 μm polycarbonate filter (Whatman, 25 mm). Prior to filtration, filters, glassware and pipette tips were rinsed three times with acid (10 mL 0.4 M HCl) and twice with sample water (10 mL). Afterwards, 20 mL of sample water was filtered and the filtrate that contained DOC was transferred to pre-combusted (4 h at 450 °C) Epa vials (40 mL). Samples were acidified with 6–7 drops of concentrated HCl (38%) to remove inorganic C and stored at 4 °C until analysis. DOC concentrations were measured using the high-temperature catalytic oxidation (HTCO) technique in a total organic C analyzer (TOC-VCPN; Shimadzu). The instrument was calibrated with a standard addition curve of Potassium Hydrogen Phthalate (0; 25; 50; 100; 200 μmol C L^−1^). Consensus Reference Materials (CRM) provided by DA Hansell and W Chen of the University of Miami (Batch 12; 2012; 41–44 μmol C L^−1^) were used as positive controls for our measurements. Concentrations measured of the batch gave average values (±SD) of 42 ± 6 μmol C L^−1^. Average analytical variation of the instrument was <3% (5–7 injections per sample). Concentrations of 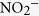 and 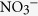 52, 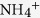 53 and 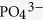 54 were analyzed using continuous flow analysis in a Quatro auto-analyzer (Seal Analytical, UK).

### Data analysis

DOC release and oxygen production rate were calculated as the change in their concentration through time. This change was solely based on the difference between initial and final concentration in case of DOC release (ESM Fig. S1), whereas for oxygen production it was based on a linear regression including all intermediate time points (ESM Fig. S2). The change of the respective control was subtracted and each rate was normalized to turf algal biomass (DW)^7^,^55^,^56^. Assumptions of heterogeneity and normality were met^57^. Differences in initial O_2_ and DOC concentrations between the treatment combinations were tested using one-way ANOVA followed by Tukey’s HSD post-hoc tests. A two-way ANOVA was used to assess whether the DOC release rates of turf algae differed among experimental treatments. All analyses were performed using software package SPSS 20 (IBM Corp., Armonk, NY, USA). As turf algae are multi-species assemblages including micro- and macroalgae, filamentous cyanobacteria and a distinct community of associated bacteria, all fluxes determined here must be considered to be net community fluxes of this turf algal holobiont (sensu Barott, *et al*.^28^).

## Results

**Figure 1:**
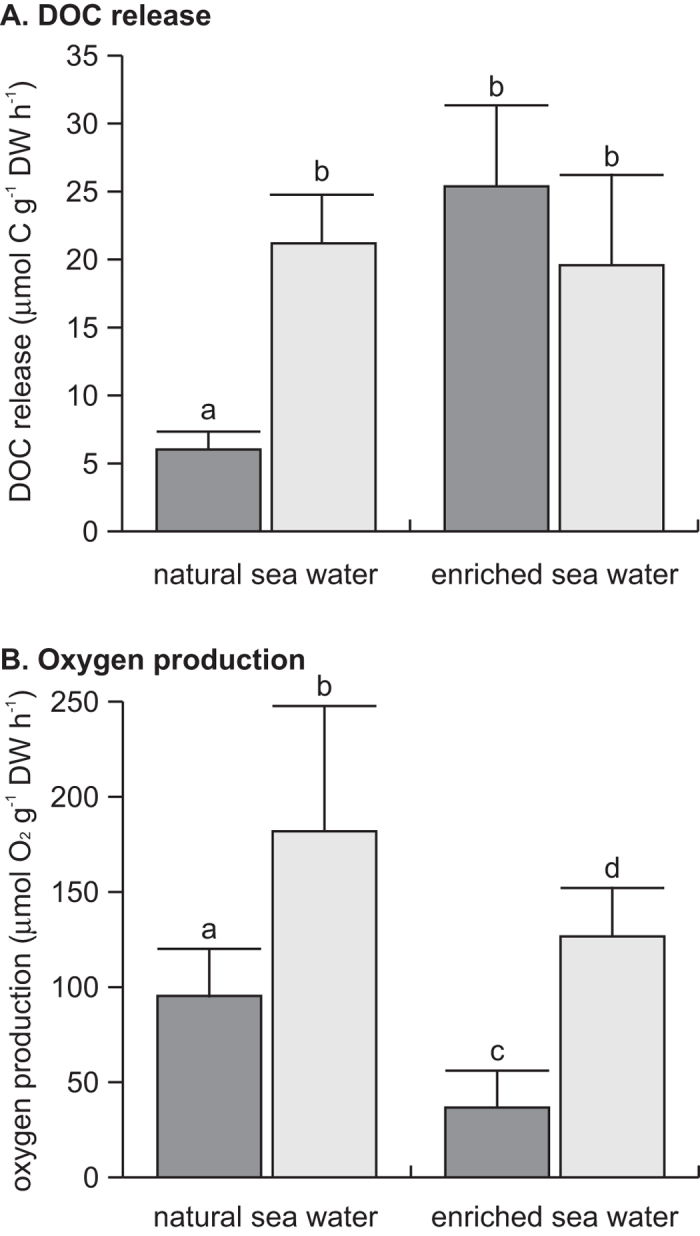
(**A**) DOC release (mean + SD) and (**B**) oxygen production (mean + SD) of turf algae for the natural and the enriched seawater treatment under reduced (dark grey) and full light conditions (light grey). In both panels n = 4 per treatment combination. Treatment combinations with the same letter are not significantly different at α = 0.05.

In natural seawater, DOC release rates were four times higher at full light than at reduced light (Tukey HSD, p = 0.012; Fig. 1A). In enriched seawater, DOC release rates at both light intensities were similar irrespective of light availability (Tukey HSD, p = 0.419), and similar to DOC release rates at full light in the natural seawater treatment (Tukey HSD, p = 0.665 and p = 0.970, respectively). A positive relation between DOC release and light availability was only observed in the natural seawater treatment, i.e. indicating that the occurrence of light-dependent DOC release depended on nutrient availability (significant interaction: light x nutrients, p = 0.002; ESM Table S3A).

Initial DOC concentrations in the incubators with turf algae and controls were 98 ± 6 μmol C L^−1^ (mean ± SD) except for the enriched seawater treatment at full light, where the DOC concentration of the control was elevated by 28 μmol C L^−1^ (Tukey HSD, p < 0.05). During the course of the incubations DOC concentrations in the controls increased by 10–12% in the natural seawater, whereas a slight decrease of −2 and −5% occurred in the enriched seawater treatments (ESM Fig. S1). In contrast, changes in DOC concentrations in the incubators with turf algae ranged between 19–57 and 22–65% in the natural and enriched seawater treatment, respectively.

Light and nutrient availability had a strong effect on oxygen production by turf algae (ESM Table S3B). Net oxygen production at full light was twice as high compared to reduced light in the natural seawater treatment (Tukey HSD p < 0.039; Fig. 1B). In contrast to DOC release, oxygen production also differed between light treatments when nutrients were added (Tukey HSD, p < 0.031). In nutrient replete treatments net oxygen production at full light was 3.5 times higher than at reduced light. Despite comparable light availability, net oxygen production under enriched conditions was 59 and 55 μmol O_2 _g^−1 ^h^−1^ DW lower in the full and reduced light treatment, respectively, compared to the natural seawater treatments (Fig. 1B). The initial oxygen concentration in the incubators with turf algae and in controls was 242 ± 16 μmol O_2 _L^−1^ (mean ± SD), except for the enriched seawater treatment at reduced light, which was 23 μmol O_2 _L^−1^ lower (Tukey HSD, p < 0.05). Changes in oxygen concentrations over time ranged between −5 and 11% in the controls, whereas the change in the incubators with turf algae ranged between 118 and 205% (ESM Fig. S2). The only exception to this general pattern occurred in the enriched seawater treatment at reduced light, where the oxygen concentration in the control changed by 45%, whereas the incubators with turf algae showed changes in oxygen concentrations between 66–74%. The net oxygen production rate of turf algae in the enriched seawater treatment at reduced light should therefore be considered with some caution.

Assuming a balanced molar ratio of carbon fixation to net oxygen production (1 mole C fixed equals 1 mole O_2_ released), 6 and 12% of the photosynthetically fixed carbon was released as DOC at reduced and full light in the natural seawater treatments. In contrast, in the enriched seawater treatments 82 and 15% of the photosynthetically fixed carbon was released as DOC at reduced and full light, respectively.

In the natural seawater treatments PO_4_^3−^ concentrations decreased by 23 and 11% during the course of the incubations at reduced and full light, respectively (Table 1). In contrast, dissolved inorganic nitrogen concentrations (DIN: 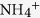 + 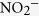 + 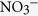) increased by 168 and 579%, respectively, indicating the release of fixed nitrogen by cyanobacteria within the turf algal communities, mainly in the form of 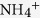. In the enriched seawater treatment at reduced light 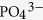 and DIN concentrations decreased by 54 and 45%, respectively, during the incubations. At full light 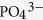 and DIN concentrations only decreased by 34 and 23%, respectively. Changes in DIN concentrations are mainly driven by the uptake of NH_4_^+^ by the turf algal community. At the end of the enriched seawater incubations PO_4_^3−^ and DIN concentrations were on average still 18 and 94 times higher than the initial concentrations in the natural seawater treatment, which suggests that both 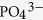 and DIN remained replete throughout the experiment. As a result of photosynthesis, the pH increased in the incubators containing turf algae by 0.55–1.07 units during the course of the experiments (Table 1). These changes are comparable to those experienced by benthic reef organisms on coral reefs over a diurnal cycle (e.g.^58^,^59^).

## Discussion

In this study we demonstrate that DOC release by turf algae increases with increasing light availability under naturally occurring nutrient concentrations. Addition of nutrients resulted in the disappearance of the positive relationship with light availability and under nutrient replete conditions DOC release became similar as in the full light and natural seawater treatment (Fig. 1A). Our results therefore indicate that the release of DOC by turf algae is affected by light and nutrient availability simultaneously.

**Figure 2:**
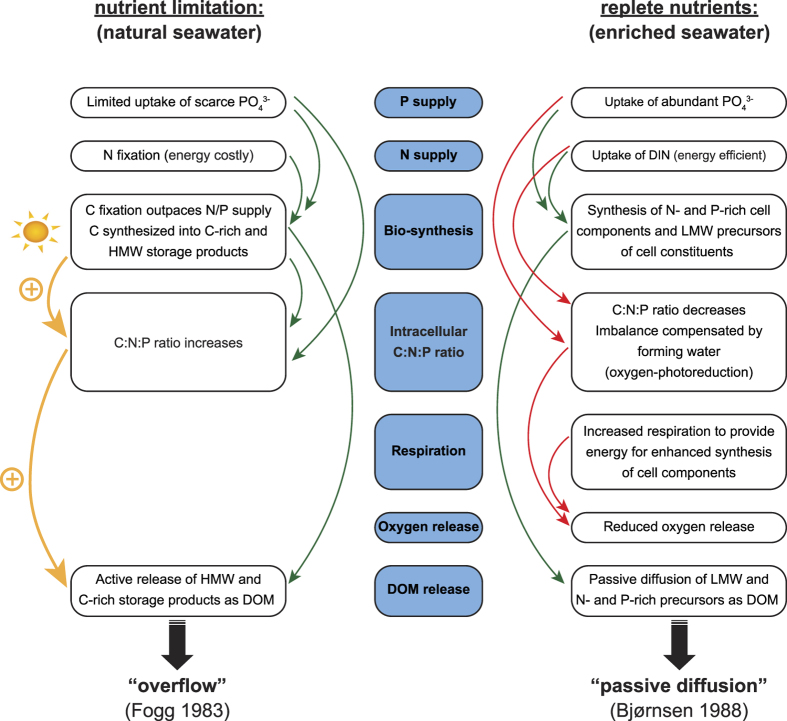
Proposed mechanisms involved in the dissolved organic matter (DOM) release of turf algae under nutrient limited and nutrient replete conditions. Under nutrient limitation carbon fixation is proposed to outpace the N and P supply, leading to the formation of C-rich and high molecular weight (HMW) storage products which are actively released as DOM in an overflow mechanism. An increase in light availability further increases the intracellular C:N:P ratio and thus stimulates DOM release. When nutrients are replete N- and P-rich cell components and low molecular weight (LMW) precursors of cell components are synthesized. These LMW molecules passively diffuse through the cell membrane as long as a concentration gradient prevails. This mechanism is not affected by an increase in light availability. A combination of increased respiration due to an enhanced synthesis and the formation of oxygen radicals and thereafter water to compensate for the imbalance of the intracellular C:N ratio are further suggested to reduce the oxygen release under such conditions. Green and red arrows indicate positive effects (stimulation) and negative effects (reduction), respectively.

Two different pathways have been proposed to explain the DOC release of aquatic primary producers: (1) A light-dependent pathway where DOC is actively released in an overflow mechanism (e.g.^37^,^38^,^39^) and (2) a light-independent pathway where DOC diffuses through the cell membrane along a concentration gradient (e.g.^42^,^43^,^44^). Light-dependent and light-independent release of DOC by turf algae were both observed in our study, but under different nutrient conditions. Thus, our results support the hypothesis that the availability of nutrients determines which pathway dominates. Under natural nutrient conditions the DOC release of turf algae was four times higher at full light compared to reduced light (Fig. 1A), confirming the reports of light-dependent DOC release by benthic reef algae and sea grasses^41^,^55^,^60^ (and references therein). Under nutrient limited conditions fixed carbon is proposed to be predominantly synthesized into carbon-rich storage products (i.e., carbohydrates, polysaccharides), as the lack of nutrients confines the synthesis of other cell components^61^,^62^ (Fig. 2). Eventually, the carbon-rich storage products are actively released as DOC in an overflow mechanism^39^,^61^,^63^. Since the carbon fixation in photosynthates is directly related to light, the resulting DOC release is also suggested to follow a positive relationship with light^37^,^40^,^64^,^65^.

Furthermore, cyanobacteria, that account for approximately 20% of the total turf algal biomass^27^,^32^,^66^,^67^, are capable of nitrogen (N_2_) fixation^32^,^68^. In fact, an increase in DIN concentrations by 5 and 13 μmol L^−1^ in natural seawater at reduced and full light, respectively, suggests nitrogen fixation and a subsequent release of excess N mainly in the form of NH_4_^+^ during the course of the experiment. As nitrogen fixation is an energy-costly process, which requires 16 ATP and 8 reduction equivalents to reduce one N_2_ molecule^69^, less energy can be allocated to carbon fixation, particularly at reduced light, and less carbon will be released as DOC. While nitrogen fixation provided turf algae with a continuous supply of N, rapid uptake of 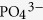 led to P depletion at the end of the natural seawater incubations (Table 1). Light-dependent DOC release, as observed in the natural seawater treatments, may therefore foremost be a result of P-limitation (e.g.^70^).

Under nutrient replete conditions DOC release rates were similar irrespective of light availability (reduced vs full light) and were comparable to those at full light of the natural seawater treatment (Fig. 1A). These findings can possibly be explained by two mechanisms (Fig. 2): Firstly, DOC release rates that do not differ under different light intensities could be explained by the ‘passive diffusion theory’ that was used as explanation for a similar phenomenon in mixed phytoplankton communities in the open ocean^43^,^44^. According to this theory, low molecular weight molecules are believed to constantly diffuse through the cell membrane as long as a concentration gradient exists^42^,^43^. And secondly, the higher DOC release rate at reduced light in nutrient enriched conditions compared to natural seawater could be explained by the ceasing of nitrogen fixation in enriched seawater. Less energy is invested in nitrogen fixation, and thus more photosynthates can be produced and eventually released as DOC than in the natural seawater treatments at similar light. In the enriched seawater treatments NH_4_^+^ concentrations decreased at both reduced and full light (Table 1). Caribbean turf algae are known to be capable of rapidly taking up 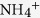 30, which suggests that the uptake of NH_4_^+^ was the predominant source of N for turf algae under nutrient replete conditions, in contrast to nitrogen fixation in the natural seawater treatments. The higher net uptake of N at reduced light in the enriched seawater treatment may further indicate a lower contribution of nitrogen fixation to the N budget compared to full light. This could compensate for the lower carbon fixation due to a lower light availability and thus, at least partly, explain similar DOC release rates at reduced and full light.

High DOC release rates by turf algae at both light treatments under nutrient replete conditions that were similar to release rates under full light in natural seawater were unexpected. This observation might however be of interest in the face of climate change, where coral reefs are increasingly subjected to run-off from land that increases nutrient availability and reduces the availability of light via suspended matter transport (e.g.^49^,^71^,^72^). Den Haan^30^ reported DIN and 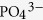 concentrations in run-off plumes coming out of the Piscadera Bay after heavy rainfall approaching those used in our enriched nutrient treatments. During such an event, DOC release rates of turf algae are therefore expected to remain high and constant, despite a reduction in light availability.

Reported DOC release rates of benthic algae vary widely between 0.14 and 5.53 mmol C m^−2^ h^−1^ and those of turf algae cover most of this spectrum (0.52 and 5.53 mmol C m^−2 ^h^−1^) (Brocke, *et al*.^8^ and references therein). When expressing our release rates per surface area, they range between 0.07 (natural seawater at reduced light) and 0.32 mmol C m^−2 ^h^−1^ (enriched seawater at reduced light) and are therefore lower than those reported in aforementioned studies. However, when our DOC release rates are normalized to DW (6.0 and 21.2 μmol C g^−1 ^h^−1^ of the natural seawater treatment with reduced and full light, respectively), they are comparable to the rates reported by Mueller, *et al*.^41^ from Curaçao (8.5 μmol C g^−1 ^h^−1^). This may indicate that the turf algal biomasses per m^2^ in our experiments were lower than in previous studies. The release of photosynthetically fixed carbon as DOC is related to the level of primary productivity^38^,^40^,^73^. Thus, DOC release is commonly expressed independent of biomass or surface area, but as a percentage of primary production. It can be assumed that 6 and 12% of the photosynthetically fixed carbon was released as DOC at reduced and full light, respectively, based on a balanced molar ratio of carbon fixation to net oxygen production in the natural seawater treatments. These percentages are within the range of reported values of coral reef benthic primary producers in previous studies: between 4^36^ and 51%^74^.

Net oxygen production increased with increasing light availability independent of nutrient conditions (Fig. 1B). However, in the enriched seawater treatments the net oxygen production of turf algae was lower than in the natural seawater treatments at comparable light intensities. Based on the dynamic energy budget^75^, this difference in net oxygen release could be explained by increased respiration under nutrient replete conditions to provide energy for the enhanced synthesis of cell compounds (e.g.^76^) (Fig. 2). Moreover, the high addition of DIN in the enriched seawater treatment is likely to have caused an imbalance in the relative abundance of C and N within the turf algae (lowered the C:N ratio). To compensate, electrons could have been donated to oxygen forming oxygen radicals , and thereafter water in the Mehler reaction^77^. This so-called oxygen-photoreduction would thus result in a lower net oxygen production. Both mechanisms imply that the DOC release in the enriched seawater treatments expressed as a percentage of net oxygen production (reduced light: 82%; full light: 15%) might be an overestimation and should therefore be considered with caution.

With this study we provide evidence that light and nutrient availability simultaneously and interactively affected the release of DOC in turf algae. The nature of the light-nutrient interaction and its influence on underlying DOC release mechanisms, which were not addressed in this study, remain to be investigated.

## Additional Information

**How to cite this article**: Mueller, B. *et al*. Effect of light and nutrient availability on the release of dissolved organic carbon (DOC) by Caribbean turf algae. *Sci. Rep.* **6**, 23248; doi: 10.1038/srep23248 (2016).

## Supplementary Material

Supplementary Information

## Figures and Tables

**Table 1:** Mean light intensities (μmol photons m^−2 ^s^−1^)(±SD) between 10:00 hrs and 16:00 hrs and mean (±SD) initial (t_0_) and final (t_end_) pH and nutrient concentrations (μmol L^−1^) for the four treatment combinations.

		Natural seawater& reduced light	Natural seawater &full light	Enriched seawater& reduced light	Enriched seawater& full light
Date		30.5.2012	30.5.2012	23.5.2012	24.5.2012
light		109 ± 44	622 ± 249	86 ± 48	585 ± 270
pH	t_0_	7.72 ± 0.15	7.53 ± 0.20	7.76 ± 0.18	7.76 ± 0.15
	t_end_	8.50 ± 0.04	8.59 ± 0.13	8.31 ± 0.13	8.59 ± 0.21
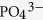	t_0_	0.019 ± 0.016	0.101 ± 0.010	11.341 ± 0.850	9.163 ± 1.517
	t_end_	0.015 ± 0.018	0.090 ± 0.122	5.249 ± 1.304	6.071 ± 0.600
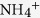	t_0_	2.831 ± 2.607	1.977 ± 1.309	69.029 ± 36.556	61.099 ± 32.825
	t_end_	7.595 ± 4.954	13.415 ± 14.147	45.705 ± 14.025	57.633 ± 6.979
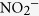	t_0_	0.129 ± 0.036	0.139 ± 0.004	0.240 ± 0.059	0.271 ± 0.170
	t_end_	0.398 ± 0.317	0.491 ± 0.378	0.297 ± 0.102	0.196 ± 0.025
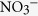	t_0_	0.434 ± 0.086	0.584 ± 0.197	3.620 ± 1.781	3.527 ± 1.767
	t_end_	0.413 ± 0.343	1.454 ± 2.199	3.278 ± 0.547	3.751 ± 0.411
