# Supplementary material for: Effect of light and nutrient availability on the release of dissolved organic carbon (DOC) by Caribbean turf algae
Source: Sci Rep. 2016 Mar 22;6:23248. doi: 10.1038/srep23248 (PMC4802385; doi:10.1038/srep23248)
Supplement: Supplementary Information [file srep23248-s1.pdf]

# Electronic supplementary materials

## Effect of light and nutrient availability on the release of dissolved organic carbon (DOC) by Caribbean turf algae

Benjamin Mueller, Joost den Haan, Petra M. Visser, Mark J.A. Vermeij, Fleur C. van Duyl

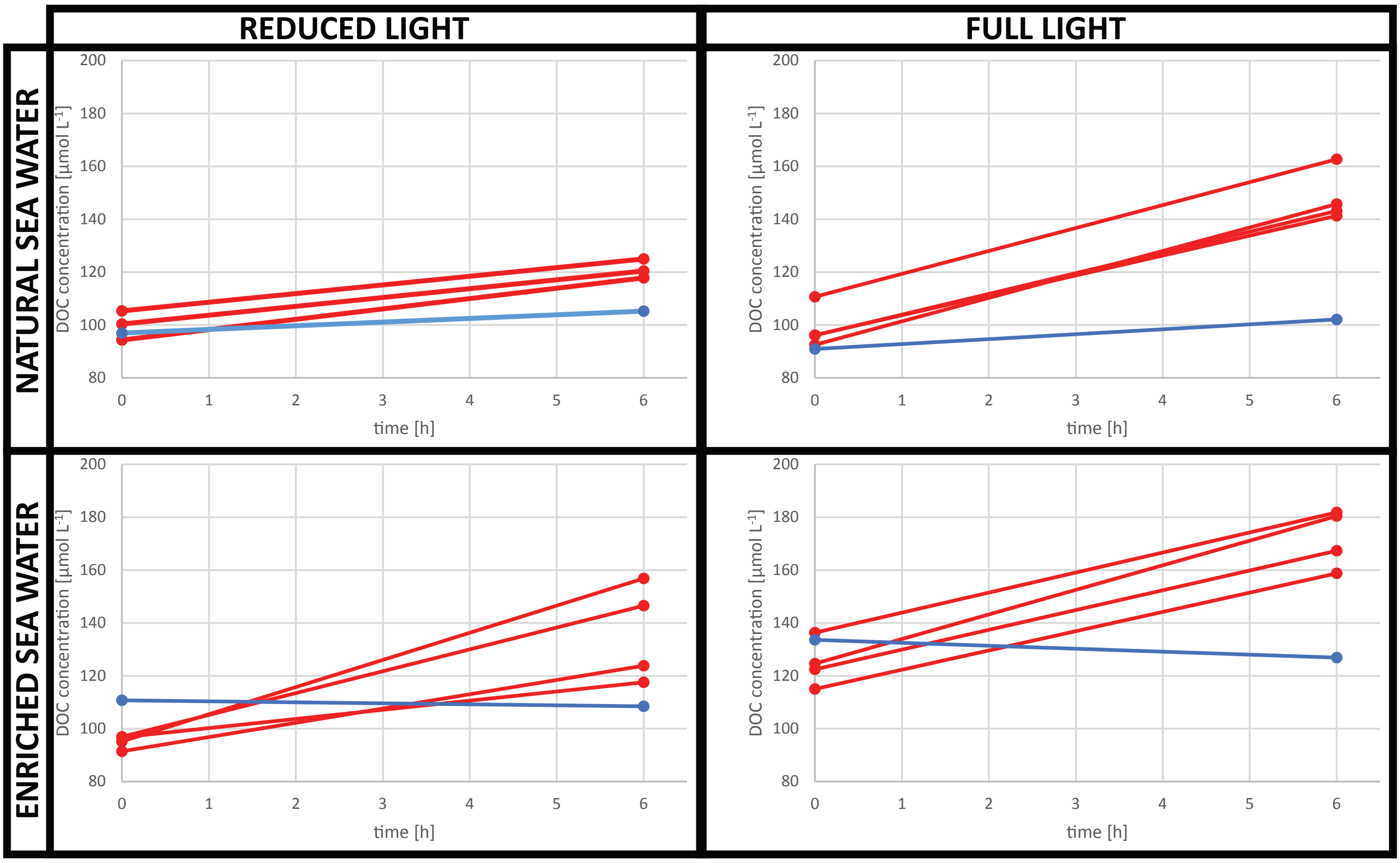


**ESM Figure S1 Change in DOC concentration through time in the four light and nutrient treatment combinations.** Each line represents an individual incubator. Incubators with turf algae are indicated in red, controls are indicated in blue.

# Electronic supplementary materials

## Effect of light and nutrient availability on the release of dissolved organic carbon (DOC) by Caribbean turf algae

Benjamin Mueller, Joost den Haan, Petra M. Visser, Mark J.A. Vermeij, Fleur C. van Duyl

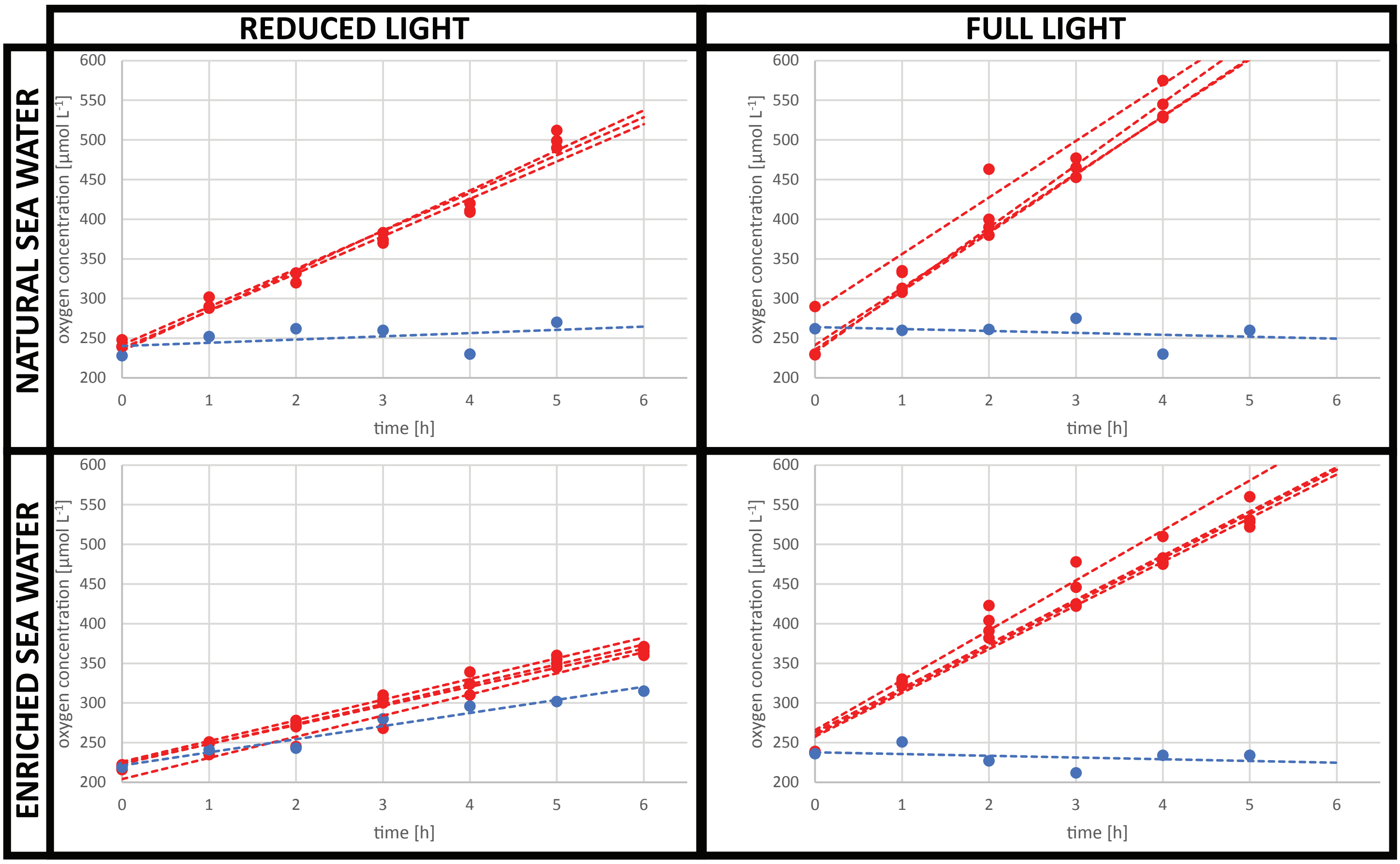


**ESM Figure S2 Change in oxygen concentration through time in the four light and nutrient treatment combinations.** Each line represents the linear regression of an individual incubator. Incubators with turf algae are indicated in red, controls are indicated in blue.

# Electronic supplementary materials

## Effect of light and nutrient availability on the release of dissolved organic carbon (DOC) by Caribbean turf algae

Benjamin Mueller, Joost den Haan, Petra M. Visser, Mark J.A. Vermeij, Fleur C. van Duyl

**ESM table S3 Results of a two-way ANOVA for the effect of light intensity and nutrient concentration on (A) DOC release and (B) oxygen production of turf algae.**

| Factor | df | F | P |
|---|---|---|---|
| **A DOC release** | | | |
| Light | 1 | 3.07 | 0.107 |
| Nutrients | 1 | 11.05 | 0.007 |
| Light × nutrients | 1 | 15.39 | 0.002 |
| **B Oxygen production** | | | |
| Light | 1 | 26.17 | <0.001 |
| Nutrients | 1 | 11.20 | 0.006 |
| Light × nutrients | 1 | 0.70 | 0.418 |
